# Factors influencing access to primary healthcare services among households in urban slums of Mogadishu, Somalia: a cross-sectional study

**DOI:** 10.1186/s12913-026-14413-5

**Published:** 2026-03-24

**Authors:** Shafie Abdulkadir Hassan, Mohammed Elmadani, Mohamed Hassan Osman, Mowlid Abdikarim Mohamed, Abdifetah Ibrahim Omar, Nur Rashid Ahmed

**Affiliations:** 1 Faculty of Medicine and Health Sciences, Jamhuriya University of Science and Technology, Mogadishu, Somalia; 2 Doctoral School of Health Sciences, Faculty of Health Sciences, University of Pécs, Vorosmarty Mihaly Street 4, Pécs, 7622 Hungary; 3 Department of Epidemiology, Faculty of Public Health, University of El Imam El Mahdi, Kosti, Sudan; 4 Jamhuriya Research Centre, Jamhuriya University of Science and Technology (JUST), Mogadishu, Somalia; 5 Advanced Research Center, Jamhuriya University of Science and Technology, Mogadishu, Somalia

**Keywords:** Access to primary healthcare, Healthcare affordability, Healthcare availability, Urban slums, Wadajir District, Logistic regression, Somalia, Universal Health coverage (UHC)

## Abstract

**Background:**

Access to primary healthcare (PHC) is essential for achieving universal health coverage (UHC), particularly in fragile and resource-limited settings such as Somalia. Urban slum populations often face multiple barriers that limit timely use of essential health services. This study assessed factors associated with household access to PHC among residents of Wadajir District in Mogadishu.

**Methods:**

A community-based cross-sectional study was conducted between October and November 2023 among 300 households selected using purposive sampling to capture underserved areas with limited health service availability. One adult household representative (≥ 18 years) was interviewed using a structured questionnaire. Descriptive and bivariate analyses were conducted using SPSS, while multivariable analysis was performed using modified Poisson regression with robust variance estimation in R to identify factors independently associated with access to PHC. Results were reported as adjusted prevalence ratios (APR) with 95% confidence intervals (CI).

**Results:**

Overall, 30% of households reported easy access to primary healthcare services. Most respondents were female (65.7%), and a large proportion reported that healthcare services were unaffordable (79.7%). In the multivariable model, respondents aged 60 years and above were significantly more likely to report easy access to PHC compared with those aged 18–29 years (APR = 2.31; 95% CI: 1.06–5.04; *p* = 0.035). Additionally, households reporting comfort with healthcare providers had significantly higher prevalence of easy access to PHC compared with those who did not feel comfortable with their providers (APR = 8.11; 95% CI: 5.33–12.33; *p* < 0.001). Other factors including sex, type of healthcare facility, waiting time, affordability of healthcare services, availability of services, and perceived quality of care were not significantly associated with access to PHC after adjustment.

**Conclusions:**

Access to primary healthcare in Wadajir District remains limited, with only one-third of households reporting easy access to services. The findings highlight the critical role of patient–provider relationships in shaping perceived access to healthcare. Interventions aimed at improving respectful communication, trust, and patient-centered care within healthcare facilities may enhance access to PHC in urban slum settings. Strengthening these interpersonal dimensions of care, alongside broader health system improvements, may help improve equitable access to healthcare.

**Supplementary information:**

The online version contains supplementary material available at 10.1186/s12913-026-14413-5.

## Introduction

Access to primary healthcare (PHC) services is a cornerstone of population health, underpinning disease prevention, early diagnosis, management of chronic conditions, and overall well-being [1]. Despite global commitments to Universal Health Coverage (UHC), substantial disparities in access to PHC persist, particularly among residents of urban slums and informal settlements [2, 3]. According to the United Nations, approximately 1.1 billion people currently live in slums worldwide, a figure projected to reach 2 billion by 2050, largely driven by rapid urbanisation in low- and middle-income countries (LMICs) [4]. Evidence from cities in Asia, Latin America, and sub-Saharan Africa consistently demonstrates that slum residents experience poorer access to essential healthcare services compared to non-slum urban populations, resulting in worse health outcomes across the life course [5, 6].

Rapid urbanisation in LMICs has contributed to the expansion of informal settlements characterised by overcrowding, inadequate infrastructure, and limited public services [7]. In these settings, access to PHC is shaped by a combination of socioeconomic constraints, service-related barriers, and individual perceptions of care [8]. Studies from urban slums in countries such as Kenya, Nigeria, India, and Bangladesh have documented persistent challenges related to healthcare affordability, long waiting times, limited availability of services, and reliance on informal providers [9–12]. These barriers contribute to delayed care-seeking, poor continuity of care, and increased preventable morbidity and mortality [9, 13, 14].

In the African context, urban slum populations face particularly pronounced barriers to PHC access due to widespread poverty, weak health systems, and limited social protection mechanisms [15, 16]. Empirical studies from Nairobi, Lagos, Accra, and Addis Ababa indicate that financial barriers, perceived poor quality of care, and overcrowded public facilities are key determinants of healthcare utilisation in informal settlements [15–19]. In addition, educational disparities and low health literacy may influence individuals’ ability to recognise health needs and navigate available services [20, 21]. These challenges are often compounded by under-resourced health facilities, shortages of trained healthcare workers, and weak referral systems, especially in rapidly growing urban areas [17, 20–22].

Somalia represents a particularly fragile context in which access to PHC remains severely constrained. Prolonged conflict, political instability, and underinvestment in public health infrastructure have contributed to one of the lowest UHC service coverage indices globally [23, 24]. Estimates suggest that only 22–27% of the population has access to essential health services, with urban poor populations disproportionately affected [25, 26]. Health indicators remain poor, including a high under-five mortality rate of 104 per 1,000 live births in 2023 [27]. Within Mogadishu, rapid urban growth, internal displacement, and the expansion of informal settlements have intensified inequities in access to basic health services, particularly for households residing in urban slums.

Although barriers to healthcare access in LMICs are well documented, empirical quantitative evidence from conflict-affected urban slum settings such as Mogadishu remains limited. Existing studies rarely quantify the relative contribution of service-related and perceived barriers to realised access to PHC in these contexts. To address this gap, the present study quantitatively examines factors associated with access to primary healthcare services among households residing in urban slums of Mogadishu, Somalia. Specifically, it assesses the association between access to PHC and facility-related characteristics (type of facility and waiting time), perceived affordability, perceived availability of services, perceived quality of care, and comfort with healthcare providers.

## Methods

### Study design and setting

This community-based cross-sectional study was conducted in Wadajir District, Mogadishu, Somalia, between October and November 2023. Wadajir is located in the south-central Banadir region and is bordered by Jazeera (Lower Shebelle region) to the south, Hodan to the west, Waberi to the north, and Dharkenley to the east [28]. As one of Mogadishu’s most densely populated urban districts, Wadajir faces significant socioeconomic and infrastructural challenges, making it an important setting for examining inequalities in access to primary healthcare.

The district also hosts a large proportion of the city’s internally displaced people (IDP) population. According to a 2012 International Committee of the Red Cross (ICRC) survey, approximately 60% of all IDP settlements and 55% of the total IDP population in Mogadishu were located in the Hodan, Wadajir, and Dharkenley districts [29, 30]. Although these figures are dated, more recent official statistics are unavailable; therefore, the 2012 estimates were used to illustrate the continued concentration of IDPs in these districts. Rapid urbanisation in Mogadishu has further expanded informal settlements that frequently lack adequate infrastructure and health services [31]. Consequently, Wadajir represents a relevant setting for examining access to healthcare among populations residing in urban slum environments.

### Sampling and participants

The study population comprised adult residents (≥ 18 years) living in households located in urban slum areas of Wadajir District, Mogadishu, regardless of whether they had previously accessed primary healthcare services. The household served as the primary sampling unit, with one adult household representative interviewed per household.

A total of 300 households were selected using purposive sampling. This non-probability sampling approach was adopted due to the absence of a reliable household sampling frame, high population mobility, and logistical and security constraints characteristic of informal urban settlements. Purposive sampling ensured the inclusion of households residing in underserved neighbourhoods with limited availability of primary healthcare services. The target sample size of 300 households was chosen to allow sufficient diversity across key socio-demographic characteristics and to support multivariable analysis, consistent with sample sizes used in comparable studies conducted in East African urban slum settings [9].

Households were identified with the assistance of local community leaders, who provided information on neighbourhood layouts and internally displaced persons (IDP) settlements to facilitate field access in areas where formal household listings were unavailable. Areas known to experience health service shortages were prioritised to capture a wide range of potential access barriers. Although this approach may have introduced selection bias by preferentially including households facing greater constraints to care, it was intentionally employed to reach hard-to-access and underserved populations that are often underrepresented in urban health research. As the study aimed to identify factors associated with access to primary healthcare rather than estimate population-level prevalence, the findings should be interpreted as reflecting conditions among vulnerable urban slum populations rather than the general population.

Within the selected neighbourhoods, trained enumerators systematically approached dwellings along mapped routes until the target sample size was reached. Each participating household was represented by one adult member (≥ 18 years) who was available at the time of the visit and who provided informed consent.

### Data collection

Data were collected through face-to-face interviews using a structured questionnaire developed specifically for this study. The instrument was designed to assess selected dimensions of access to primary healthcare, alongside relevant socio-demographic characteristics.

The questionnaire was initially developed in English, reviewed by three public health experts for clarity and content validity, translated into Somali, and back-translated to ensure linguistic accuracy. The instrument was pilot tested among 20 participants to refine unclear items. Reliability testing was conducted for multi-item scales, with Cronbach’s alpha values exceeding 0.7.

Participants were asked to base their responses on their most recent healthcare interaction within the previous 12 months to minimise recall bias. The questionnaire collected information on socio-demographic characteristics, including sex, age, education level, marital status, and household size. Access to primary healthcare was assessed using self-reported measures reflecting perceived access to services. The primary outcome variable was respondents’ perception of whether it was easy for their household to access primary healthcare services when needed.

Additional variables included the type of healthcare facility usually visited, waiting time before receiving care, perceived affordability of healthcare services, perceived availability of healthcare services in the respondent’s area, overall perceived quality of care received, and comfort in discussing health concerns with healthcare providers. Affordability and availability were measured using single-item questions reflecting respondents’ perceptions rather than objective financial or structural indicators.

Respondents were also asked to identify perceived barriers to accessing primary healthcare and to suggest potential improvements, such as reducing waiting times, lowering healthcare costs, and improving service availability. A summary of questionnaire items and response options is provided in the supplementary appendix (S1) to enhance transparency and reproducibility.

### Statistical analysis

Data were analysed using the Statistical Package for the Social Sciences (SPSS), version 27, and R statistical software (version 4.5.2). Descriptive statistics were conducted in SPSS to summarize participants’ socio-demographic characteristics and healthcare-related variables. As all variables were categorical, results were presented as frequencies and percentages.

Bivariate analysis was performed using the Pearson chi-square (χ²) test in SPSS to examine the association between access to primary healthcare services and independent variables. These variables included socio-demographic characteristics (age group, sex, marital status, education level, and household size), healthcare utilization characteristics (frequency of healthcare visits and type of healthcare facility), and perceived healthcare service factors (waiting time, affordability of healthcare services, availability of healthcare services, perceived quality of care, and comfort with healthcare providers).

Variables with a p-value < 0.20 in the bivariate analysis, as well as variables considered conceptually relevant [32], were included in the multivariable modified Poisson regression model with robust variance estimation following recommendations for purposeful variable selection in regression modeling, which was conducted using R statistical software. This approach was selected because the outcome variable had a relatively high prevalence, making prevalence ratios more appropriate than odds ratios for cross-sectional studies. Results of the regression analysis were reported as adjusted prevalence ratios (APR) with 95% confidence intervals (CI). Statistical significance was defined as *p* < 0.05.

## Results

A total of 300 respondents were included in the study (Table 1). Nearly half of the participants were aged 30–44 years (49.3%), while 26.7% were aged 45–59 years, 13.0% were aged 60 years or older, and 11.0% were aged 18–29 years. Most respondents were female (65.7%). Regarding marital status, 59.0% were divorced/widowed, 36.3% were married, and 4.7% were single. More than half of the participants had primary education (57.3%), while 39.0% had no formal education and 3.7% had secondary or higher education. In terms of household size, 41.7% lived in households with seven or more persons, 40.0% in households with 1–4 persons, and 18.3% in households with 5–6 persons. Regarding healthcare utilization, 41.3% reported low utilization (0–1 visit), 39.0% moderate utilization (2–3 visits), and 19.7% high utilization (≥ 4 visits).

**Table 1:** Demographic and household characteristics of study participants (*N* = 300)

Variable	Category	*n*	%
Age	18–29 years	33	11
	30–44 years	148	49.3
	45–59 years	80	26.7
	60 + years	39	13
Sex	Male	103	34.3
	Female	197	65.7
Marital status	Married	109	36.3
	Divorced/Widowed	177	59
	Single	14	4.7
Education status	No formal education	117	39
	Primary	172	57.3
	Secondary/Higher	11	3.7
Household size	1–4 persons	120	40
	5–6 persons	55	18.3
	≥ 7 persons	125	41.7
Frequency of healthcare visits	0–1 visit (low utilization)	124	41.3
	2–3 visits (moderate utilization)	117	39
	≥ 4 visits (high utilization)	59	19.7

**Table 2:** Healthcare service characteristics and perceptions of respondents (*N* = 300)

Variable	Category	*n*	%
Type of healthcare facility usually visited	Public	139	46.3
	Private	161	53.7
Waiting time before receiving care	< 30 min	113	37.7
	30–60 min	187	62.3
Affordability of healthcare services	No	239	79.7
	Yes	61	20.3
Availability of healthcare services in the area	Inadequate availability	103	34.3
	Adequate availability	197	65.7
Perceived quality of care in hospitals	Poor quality	245	81.7
	Good quality	55	18.3
Comfort with healthcare providers	No	212	70.7
	Yes	88	29.3
Main barriers to accessing PHC services	Cost of services	262	87.3
	Long waiting time	23	7.7
	Limited-service availability	15	5
Changes that would improve PHC access	Reduced cost	139	46.3
	Closer facilities	65	21.7
	Shorter waiting time	84	28
	Better service availability	12	4
Ease of household access to PHC	No	210	70
	Yes	90	30

Regarding healthcare service characteristics (Table 2), more than half of the respondents reported visiting private healthcare facilities (53.7%), while 46.3% reported using public facilities. Most respondents reported waiting 30–60 min before receiving care (62.3%). A large proportion of participants indicated that healthcare services were not affordable (79.7%). Approximately two-thirds of respondents reported adequate availability of healthcare services in their area (65.7%). However, the majority perceived the quality of care in hospitals as poor (81.7%) and reported not feeling comfortable with their healthcare providers (70.7%). The most frequently reported barrier to accessing primary healthcare services was the cost of services (87.3%). Nearly half of respondents indicated that reducing healthcare costs would most improve access to primary healthcare (46.3%). Overall, 70.0% of households reported difficulty accessing primary healthcare services.

**Table 3:** Bivariate association between study variables and access to primary healthcare services (Chi-square test)

Variable	Category	Easy Access *n* (%)	Difficult Access *n* (%)	χ²	*p*-value
Age	18–29 years	4 (12.1)	29 (87.9)	6.92	0.075
	30–44 years	50 (33.8)	98 (66.2)		
	45–59 years	22 (27.5)	58 (72.5)		
	≥ 60 years	14 (35.9)	25 (64.1)		
Sex	Male	23 (22.3)	80 (77.7)	4.39	**0.036**
	Female	67 (34.0)	130 (66.0)		
Marital status	Married	26 (23.9)	83 (76.1)	3.12	0.211
	Divorced/Widowed	59 (33.3)	118 (66.7)		
	Single	5 (35.7)	9 (64.3)		
Education status	No formal education	30 (25.6)	87 (74.4)	1.91	0.386
	Primary	57 (33.1)	115 (66.9)		
	Secondary/Higher	3 (27.3)	8 (72.7)		
Household size	1–4 persons	32 (26.7)	88 (73.3)	1.71	0.426
	5–6 persons	20 (36.4)	35 (63.6)		
	1–4 persons	32 (26.7)	88 (73.3)		
Frequency of healthcare visits	0–1 visit	36 (29.0)	88 (71.0)	0.53	0.767
	2–3 visits	34 (29.1)	83 (70.9)		
	≥ 4 visits	20 (33.9)	39 (66.1)		
Type of healthcare facility	Public	45 (32.4)	94 (67.6)	0.7	0.404
	Private	45 (28.0)	116 (72.0)		
Waiting time	< 30 min	33 (29.2)	80 (70.8)	0.06	0.815
	30–60 min	57 (30.5)	130 (69.5)		
Affordability of services	No	72 (30.1)	167 (69.9)	0.01	0.925
	Yes	18 (29.5)	43 (70.5)		
Availability of services	Inadequate	28 (27.2)	75 (72.8)	0.59	0.442
	Adequate	62 (31.5)	135 (68.5)		
Quality of care	Poor	75 (30.6)	170 (69.4)	0.24	0.625
	Good	15 (27.3)	40 (72.7)		
Comfort with providers	No	21 (9.9)	191 (90.1)	**138.96**	**< 0.001**
	Yes	69 (78.4)	19 (21.6)		

Bivariate analysis using the Pearson chi-square test showed that sex and comfort with healthcare providers were significantly associated with household access to primary healthcare services (Table 3). Female respondents were more likely to report easy access to primary healthcare compared with males (*p* = 0.036). Comfort with healthcare providers was strongly associated with access to services (*p* < 0.001). Age showed a borderline association with access to primary healthcare (*p* = 0.075), while other variables including marital status, education level, household size, healthcare utilization, facility type, waiting time, affordability, availability, and perceived quality of care were not significantly associated with access.

**Table 4:** Factors associated with household access to primary healthcare services (Modified Poisson regression)

Variable	APR	95% CI	*p*-value
**Age**			
18–29 years	Reference		
30–44 years	1.85	0.79–4.34	0.155
45–59 years	2.05	0.88–4.78	0.098
60 + years	**2.31**	**1.06–5.04**	**0.035**
**Sex**			
Male	Reference		
Female	1.07	0.71–1.62	0.742
**Type of healthcare facility**			
Public	Reference		
Private	1.1	0.81–1.49	0.544
**Waiting time**			
< 30 min	Reference		
30–60 min	1.18	0.83–1.69	0.35
**Affordability**			
Not affordable	Reference		
Affordable	0.93	0.68–1.26	0.637
**Availability of services**			
Inadequate	Reference		
Adequate	1.03	0.79–1.34	0.826
**Quality of care**			
Poor quality	Reference		
Good quality	1.36	0.83–2.23	0.228
**Comfort with healthcare providers**			
No	Reference		
Yes	**8.11**	**5.33–12.33**	**< 0.001**

Note: ***APR***: *Adjusted Prevalence Ratio*, ***CI***: *Confidence Interval*

Modified Poisson regression with robust variance estimation was used to identify factors associated with household access to primary healthcare services (Table 4). After adjustment for potential confounders, respondents aged 60 years or older had significantly higher prevalence of reporting easy access to primary healthcare compared with those aged 18–29 years (APR = 2.31, 95% CI: 1.06–5.04, *p* = 0.035). In addition, households reporting comfort with healthcare providers were significantly more likely to report easy access to primary healthcare services compared with those who did not feel comfortable with their providers (APR = 8.11, 95% CI: 5.33–12.33, *p* < 0.001). Other factors, including sex, type of healthcare facility, waiting time, affordability of healthcare services, availability of services, and perceived quality of care, were not significantly associated with access to primary healthcare in the adjusted model.

## Discussion

This study examined factors associated with household access to primary healthcare (PHC) services among residents of urban slums in Mogadishu, Somalia. Overall, most households reported that access to PHC was not easy, suggesting persistent inequities in healthcare access within this highly vulnerable urban population. Access to healthcare is widely recognized as a multidimensional concept that includes the availability, accessibility, affordability, accommodation, and acceptability of services [33]. In many urban slum settings in low- and middle-income countries (LMICs), these dimensions interact with social and economic vulnerabilities to create complex barriers to healthcare utilization. Consistent with this framework, previous studies have shown that residents of informal settlements often face overlapping structural, financial, and social barriers that limit their ability to access essential health services [34]. For example, a study conducted in Nairobi’s urban slums [9] similarly reported poor access to PHC, while a recent scoping review of slum health literature [17] concluded that healthcare access and utilization in such settings are shaped by the interaction of household vulnerability, service organization, and socioeconomic constraints. In Somalia, these challenges occur within a fragile health system characterized by limited health infrastructure and severe workforce shortages, further constraining access to primary healthcare services [35].

The most important finding of this study was the very strong association between comfort with healthcare providers and easier access to PHC. Households reporting comfort with providers had substantially higher prevalence of easy access compared with those who did not. This finding is consistent with growing evidence that interpersonal quality of care and patient–provider relationships play a crucial role in healthcare utilization. Effective communication between patients and healthcare providers has been shown to improve patient satisfaction, trust, and adherence to medical advice, ultimately influencing healthcare-seeking behavior and service utilization [36, 37]. A recent study conducted in rural China [38] found that better provider–patient communication was positively associated with perceived healthcare quality and patient satisfaction, while a study in Ethiopia demonstrated that perceived quality of care, particularly provider communication and information provision, was associated with increased use of nearby health centers [39]. Together, these findings support the interpretation that when patients feel respected, listened to, and understood by healthcare providers, they are more likely to perceive services as accessible and continue using them. In resource-constrained settings such as urban slums in Mogadishu, where structural barriers to healthcare are already substantial, trust and positive interactions with healthcare providers may become especially important determinants of perceived access.

This finding also highlights the importance of acceptability, one of the key dimensions of healthcare access described in classic access frameworks [33]. Even when healthcare services are geographically available, individuals may still perceive access as limited if interactions with healthcare providers are uncomfortable, disrespectful, or characterized by poor communication. Similar observations have been reported in other slum contexts, where patients’ experiences with healthcare providers strongly influence their willingness to seek care [34]. The Nairobi slum study [9], for instance, emphasized that healthcare access is shaped not only by financial or geographic barriers but also by how individuals experience care within health facilities. The present findings extend this understanding by demonstrating that the interpersonal dimension of care may be a particularly important determinant of perceived healthcare access in fragile urban environments.

Another important finding was that respondents aged 60 years and older were more likely to report easy access to PHC compared with younger adults aged 18–29 years. One possible explanation is that older adults often have greater healthcare needs due to the higher prevalence of chronic diseases and age-related conditions, which may increase their interaction with healthcare systems. Greater familiarity with healthcare services and providers may also facilitate access among older individuals. However, this finding should be interpreted cautiously, as the broader literature on healthcare access in LMICs frequently highlights older age as a potential source of vulnerability rather than advantage. A recent scoping review examining older adults’ access to PHC in LMICs [40] found that older populations commonly face multiple barriers to healthcare, including financial constraints, transportation challenges, and limited social support. The positive association observed in this study may therefore reflect local patterns of healthcare utilization or health needs rather than inherently better access conditions for older populations.

Although sex was associated with access in the bivariate analysis, this association was no longer significant after adjustment in the multivariable model. This suggests that differences observed at the descriptive level may be explained by other factors, particularly perceptions of healthcare services and interactions with providers. The literature on sex differences in healthcare utilization in slum settings is mixed. Some studies have reported higher healthcare utilization among women, partly due to reproductive health needs, while others have identified gender-related barriers to healthcare access, including financial dependence and sociocultural constraints [17]. In the Nairobi slum study [9], for example, female-headed households were found to experience lower access to healthcare services. However, that study measured the sex of the household head rather than the sex of the individual respondent, which may partly explain differences in findings. The present study suggests that, within this context, sex alone may not independently determine access to PHC once other service-related factors are considered.

An interesting finding of this study is that affordability was not independently associated with easier access to PHC in the adjusted model, even though a large majority of respondents reported that healthcare services were unaffordable. This result appears to differ from much of the global literature, which identifies financial barriers as a key determinant of healthcare utilization in LMICs. Previous studies have shown that high out-of-pocket payments and limited financial protection can significantly restrict access to healthcare services, particularly among poor populations [41, 42]. One possible explanation for the present finding is that financial hardship may be widespread across the study population, resulting in limited variation in affordability between households. In such situations, affordability may represent a universal constraint rather than a factor that differentiates households with better or worse access to healthcare. This interpretation is consistent with broader evidence from slum health research, which highlights income insecurity, unstable employment, and competing livelihood priorities as common barriers to healthcare utilization among urban poor populations [17, 34].

Similarly, type of healthcare facility, waiting time, perceived availability of services, and perceived quality of care were not significantly associated with access to PHC in the multivariable model. This does not necessarily imply that these factors are unimportant. Instead, it may indicate that their influence is either weaker than the interpersonal dimension of care or closely correlated with other perceptions of healthcare services. Previous research has shown that perceived quality of care can influence healthcare utilization, particularly when it reflects provider communication, respect, and responsiveness to patient needs [37, 39]. In the context of this study, the strong association between comfort with healthcare providers and perceived access suggests that the interpersonal component of care may capture a substantial portion of how households evaluate their ability to access healthcare services.

The descriptive findings on perceived barriers and suggested improvements provide additional insights into community perspectives on healthcare access. Cost of services was overwhelmingly identified as the primary barrier, and reducing healthcare costs was the most frequently suggested improvement, followed by shorter waiting times and closer facilities. These perceptions are consistent with findings from other urban slum settings, where both direct and indirect costs of seeking care, including transportation expenses and lost income from time away from work, often influence healthcare-seeking decisions [34]. Although these factors were not independently associated with access in the adjusted model, they remain highly relevant for health policy and program design because they reflect the lived experiences of households in these communities.

Taken together, the findings of this study suggest that improving access to primary healthcare in urban slums in Mogadishu will require more than expanding the physical availability of healthcare facilities. Strengthening the interpersonal quality of care, including respectful treatment, effective communication, and patient-centered engagement, may play a critical role in improving perceived access to healthcare services. This perspective aligns with broader global health evidence emphasizing that improving health system performance requires not only expanding service coverage but also ensuring that healthcare services are responsive to the needs and expectations of patients [35, 39, 43]. In fragile health system contexts such as Somalia, interventions aimed at strengthening provider–patient communication and building trust within communities may represent a relatively feasible and high-impact strategy for improving access to primary healthcare services.

## Implications for policy and practice

The findings of this study have several important implications for improving access to primary healthcare in urban slum settings in Somalia. First, the strong association between comfort with healthcare providers and access to PHC highlights the importance of interpersonal quality of care as a critical component of healthcare accessibility. While health policies often focus on expanding infrastructure or increasing service availability, the results suggest that patient–provider relationships and communication play a crucial role in shaping perceptions of healthcare access. Strengthening respectful care, improving communication skills among healthcare workers, and promoting patient-centered approaches could enhance trust and encourage healthcare utilization among underserved populations.

Second, although affordability was widely reported as a barrier to accessing healthcare services, it was not independently associated with access in the multivariable model. This finding suggests that financial constraints may be pervasive across the study population, affecting most households similarly. Nevertheless, the high proportion of respondents reporting unaffordable services indicates the continued need for financial protection mechanisms, such as subsidized healthcare services, community-based health insurance schemes, or targeted support for vulnerable households. Such interventions could reduce out-of-pocket expenditures and improve equitable access to PHC services.

Third, the finding that older adults were more likely to report easier access to PHC may reflect greater health needs and familiarity with the healthcare system among this group. However, it also suggests that younger adults may face additional barriers to healthcare utilization, including competing livelihood demands or lower perceived need for care. Public health programs aimed at improving healthcare access in urban slum populations should therefore consider strategies that address barriers faced by different age groups.

Finally, the descriptive findings on perceived barriers indicate that cost of services, waiting time, and service availability remain key concerns among residents. Policymakers and health system planners should prioritize strengthening primary healthcare services within informal settlements, including improving facility accessibility, reducing waiting times, and ensuring consistent availability of essential services. Addressing these issues could help improve both the perceived and actual accessibility of PHC services in vulnerable urban communities.

## Limitations and future research

This study has several limitations that should be considered when interpreting the findings. First, the cross-sectional design limits the ability to establish causal relationships between the examined factors and access to primary healthcare services. As a result, the direction of association cannot be determined with certainty. For example, while comfort with healthcare providers was strongly associated with easier access to PHC, it is also possible that individuals who access healthcare more frequently develop greater comfort and familiarity with providers.

Second, the study relied on self-reported measures of healthcare access and perceptions of healthcare services, which may be subject to recall bias or social desirability bias. Participants were asked to base their responses on their most recent healthcare experience within the previous 12 months; however, inaccuracies in recall may still have occurred.

Third, the use of purposive sampling may limit the generalizability of the findings beyond the specific urban slum communities included in the study. While this approach was necessary due to the absence of a reliable household sampling frame and the logistical constraints of working in informal settlements, it may have introduced selection bias. Therefore, the findings should be interpreted as reflecting conditions among vulnerable populations in Wadajir District rather than the broader population of Mogadishu or Somalia.

Fourth, several key variables in the study were measured using single-item perception-based questions, including affordability, availability, and quality of care. Although such measures are commonly used in health services research, they may not fully capture the complexity of healthcare access or service quality.

Future research should consider using larger and more representative samples, as well as mixed-methods approaches that combine quantitative surveys with qualitative interviews. Qualitative research could provide deeper insights into the social, cultural, and institutional factors that influence healthcare access in urban slum settings. In addition, longitudinal studies would help clarify the causal pathways linking patient–provider relationships, service characteristics, and healthcare utilization. Further research examining health system factors such as workforce capacity, facility distribution, and financial protection mechanisms would also contribute to a more comprehensive understanding of barriers to primary healthcare access in Somalia.

## Conclusion

This study examined factors associated with perceived access to primary healthcare among households residing in selected urban slum communities in Wadajir District, Mogadishu. Within this study sample, only a minority of households reported easy access to primary healthcare services. Comfort with healthcare providers was strongly associated with easier access to PHC, suggesting that interpersonal aspects of care, including respectful communication and patient–provider trust, may play an important role in shaping how households experience healthcare access. Older respondents were also more likely to report easier access to services, while other factors such as affordability, waiting time, and facility type were not independently associated with access after adjustment. These findings should be interpreted as reflecting associations observed within this vulnerable cohort rather than representing the broader population of urban slum residents in Mogadishu. Nevertheless, the results highlight the potential importance of strengthening patient-centered care and provider–patient communication within primary healthcare services serving underserved urban communities.

## Supplementary Information

Below is the link to the electronic supplementary material.

Supplementary Material 1

## Data Availability

The datasets used and/or analysed during the current study are available from the corresponding author upon reasonable request.
